# Squamous cell carcinoma of renal pelvis and percutaneous nephrolithotomy tracts after PCNL for staghorn calculus - point of surgical technique

**DOI:** 10.3332/ecancer.2025.1900

**Published:** 2025-04-25

**Authors:** Singamaneni Arun Mitra, Vijith Shetty, Achuth S Nayak, Anand Raja

**Affiliations:** 1 Department of Surgical Oncology, Cancer Institute (WIA), 38, Sardar Patel Road, Chennai 600036, Tamil Nadu, India; 2 Medical Oncology, KS Hegde Medical Academy, Mangalore 575018, Karnataka, India; 3 Radiation Oncology, Zulekha Yenepoya Institute Oncology, Deralakatte 575018, Karnataka, India; a https://orcid.org/0000-0001-5843-3667; b https://orcid.org/0000-0002-2585-8527

**Keywords:** nephrostomy, percutaneous, nephrectomy, carcinoma, squamous cell, kidney calculi, radical surgery

## Abstract

**Introduction:**

Squamous cell carcinoma (SCC) comprises 0.5%–15% of tumours in the renal pelvis and ureter. SCC after percutaneous nephrolithotomy (PCNL) for staghorn calculus is a rare entity with only five cases reported. Tumour spreading along the tract after PCNL is even more uncommon with only one case reported. We report a case of SCC of the renal pelvis and tract of PCNL extending upto skin over the flank and review the literature, demonstrate surgical technique.

**Case report:**

A 54-year-old gentleman was diagnosed with right pelvic staghorn lithiasis due to flank pain and confirmed on a non-contrast computed tomography (CT) scan. He underwent PCNL in two stages over 2 weeks apart. There was no suspicious lesion after the complete removal of the stone. Due to persistent right flank pain and hematuria after 3 months, the patient was evaluated with a contrast CT and magnetic resonance imaging (MRI) which revealed an enhancing lesion over the right kidney extending from the renal pelvis to the PCNL tract associated with retrocaval, aortocaval and precaval nodes. CT-guided biopsy of the mass was performed diagnosing a high-grade carcinoma with squamous differentiation. Urine cytology showed dysplastic cells. Diethylenetriaminepentaacetic acid study revealed a low glomerular filtration rate of 20 mL/min in the right kidney. There were no metastases elsewhere. We performed radical nephrectomy along with excision of the PCNL tracts, skin and flank muscles excision with template-based retroperitoneal lymph node dissection. Finally, we use a mesh for reconstruction.

**Conclusion:**

Long-standing staghorn calculus may harbor SCC of the renal pelvis which is undiagnosed preoperatively probably due to chronic irritation. Complete surgical excision with negative margins (R0) is the only option to cure as demonstrated in this case.

## Introduction

Urothelial cancer stands as the most prevalent form of malignancy affecting the upper urinary tract, with squamous cell carcinoma (SCC) comprising a small yet noteworthy subset, accounting for 0.5% to 15% of tumours [1]. Established risk factors for SCC include chronic pyelonephritis and nephrolithiasis. Notably, staghorn calculus, characterised by its large size and occupation of the renal pelvis with branching into calyces, has been associated with an increased risk of developing SCC [2]. Despite its rarity, the manifestation or development of SCC post-percutaneous nephrolithotomy (PCNL) for staghorn calculus represents an unusual occurrence, with only five cases documented in the literature [2–6] (Table 1). Furthermore, the extension of the tumour to the PCNL tract is an exceedingly rare phenomenon, with only one case reported to date [3]. Here, we present a case of SCC originating from the renal pelvis, with the tumour extending along the PCNL tract involving the skin over the flank and the surgical technique to deal with such cases.

## Case report

**Figure 1.**
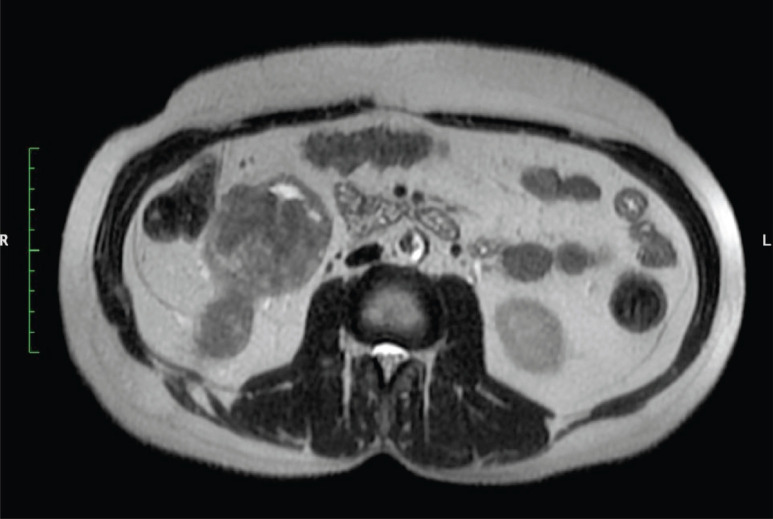
MRI abdomen axial T2 image reveal right lower pole renal tumour with tract of PCNL.

**Figure 2.**
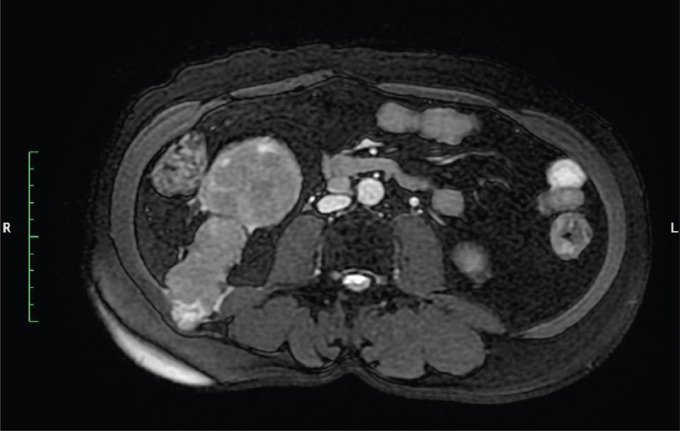
MRI abdomen axial T2 FATSAT image show tract of PCNL from lower pole kidney to abdominal muscles.

**Figure 3.**
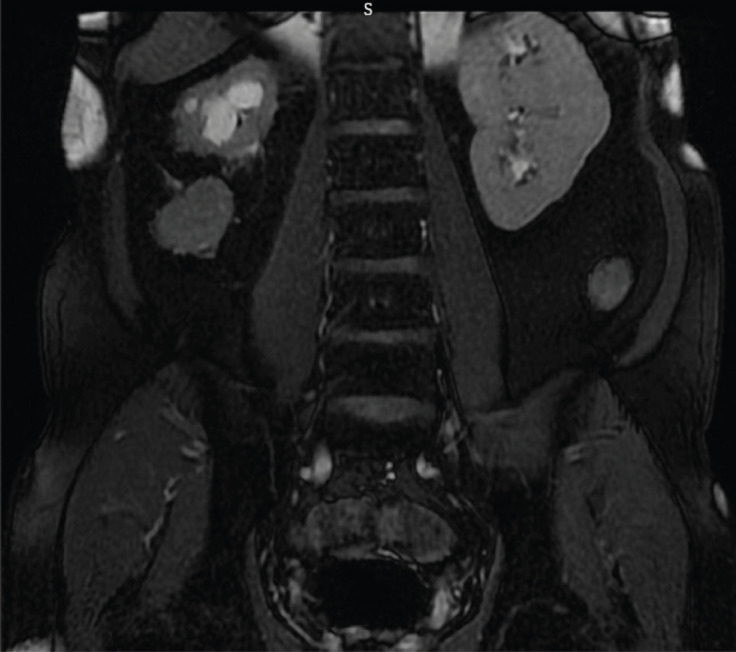
Coronal T2 image of MRI abdomen reveals the tract around right kidney.

**Figure 4.**
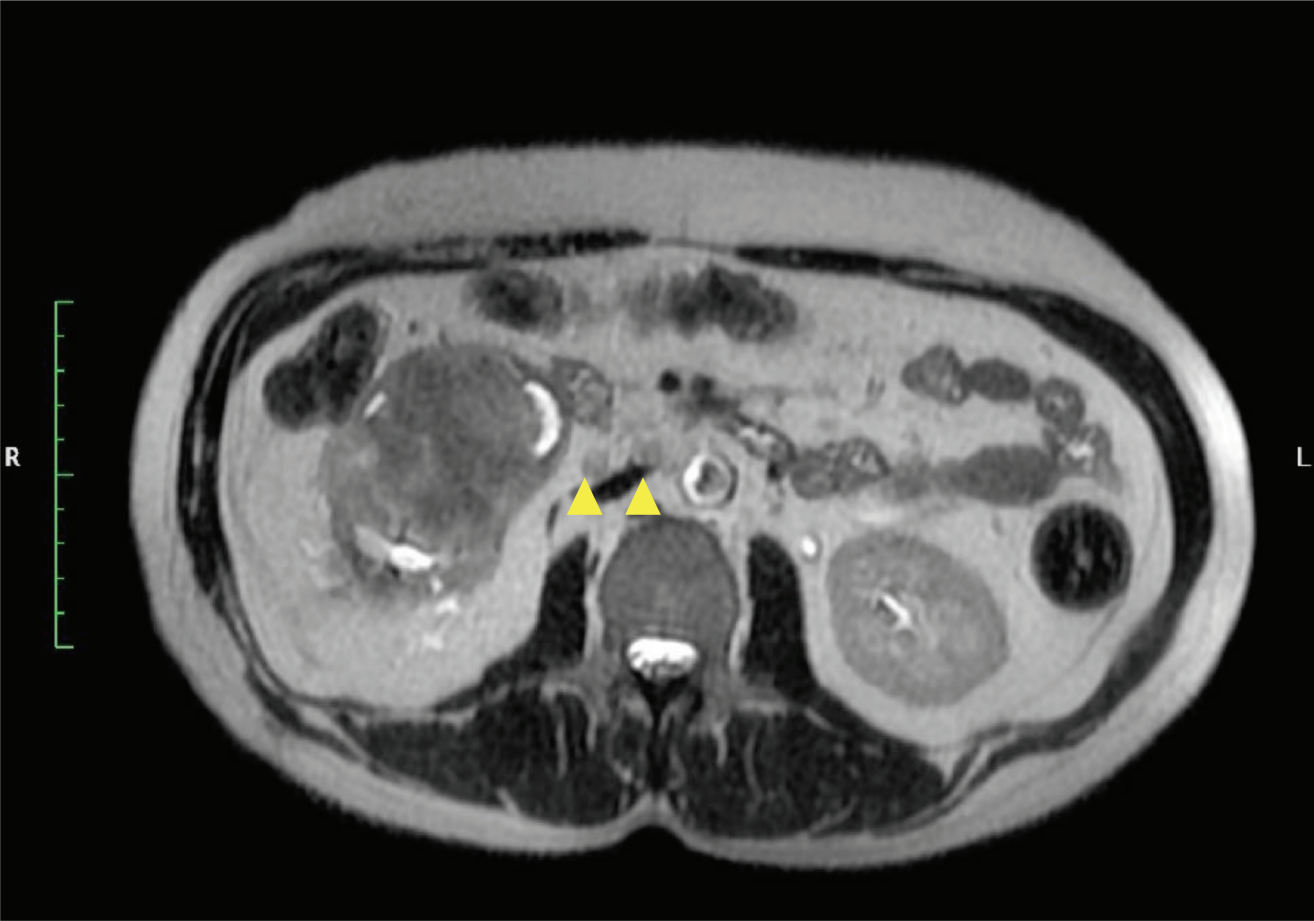
MRI T2 axial image show right renal hilar nodes (yellow arrow heads).

A 54-year-old gentleman without any comorbidity, presented with right flank pain and was diagnosed with a staghorn calculus in the renal pelvis that was confirmed on non-contrast computed tomography (CT) without any suspicious mass. Subsequently, the patient underwent two PCNLs until the lithiasis was completely removed. After 3 months, the patient continued with persistent right flank pain and occasional hematuria so was referred to our institute. Further evaluation via contrast CT and magnetic resonance imaging (MRI) revealed an enhancing lesion within the right kidney, extending from the renal pelvis to the PCNL tract associated with enlarged retrocaval, aortocaval and precaval nodes (Figures 1–4). A CT-guided biopsy confirmed a high-grade carcinoma with squamous differentiation. Blood urea was 30 mg/dL, Creatinine - 1.2 mg/dL, Calcium - 9.2 mg/dL and Phosphate - 3 mg/dL. Urine cytology revealed dysplastic cells, while a diethylenetriaminepentaacetic acid study indicated a reduced glomerular filtration rate of 20 mL/min of the affected kidney, with no evidence of metastases elsewhere. Consequently, the patient underwent radical nephrectomy with en bloc excision of the two PCNL tracts, the involved skin and the flank muscles with template-based retroperitoneal lymph node dissection, followed by mesh reconstruction. The postoperative period was uneventful. The final histopathological finding demonstrated a kidney measuring 15 × 8 × 8 cm, a 12 cm ureter, along with two separate pieces of skin containing underlying PCNL tracts measuring 8 × 3.5 × 5 and 5 × 2.5 × 2 cm, respectively. Microscopic examination demonstrated tumour infiltration from the renal pelvis extending through the perirenal fat into the PCNL tract up to the skin, with histologic features indicative of SCC grade 2. All resected margins were free of tumour, with no evidence of lymphovascular or perineural invasion. Furthermore, 29 dissected lymph nodes were devoid of metastases. The patient received adjuvant radiation covering tumour bed, paraaortic nodal region to a dose of 50.4 Gy in 28 fractions. Further, chemotherapy was given Weekly with Paclitaxel 80 mg/m^2^ and Carboplatin (AUC 2) for 12 weeks. The patient remained disease-free after 2 years of follow-up.

To the best of our knowledge, only one such case has been previously reported, wherein the dissemination of SCC originating from the renal pelvis involving PCNL tracts and reaching the skin.

### Operative technique

Surgery was performed under general and epidural anesthesia.

#### Step 1

**Figure 5.**
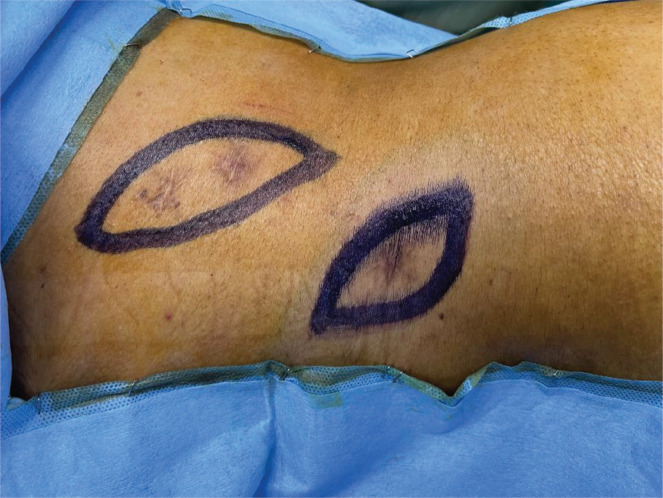
Patient was in semi-prone position. Scars of PCNL tracts were seen. Incision was marked around the scars with 1 cm margin.

**Figure 6.**
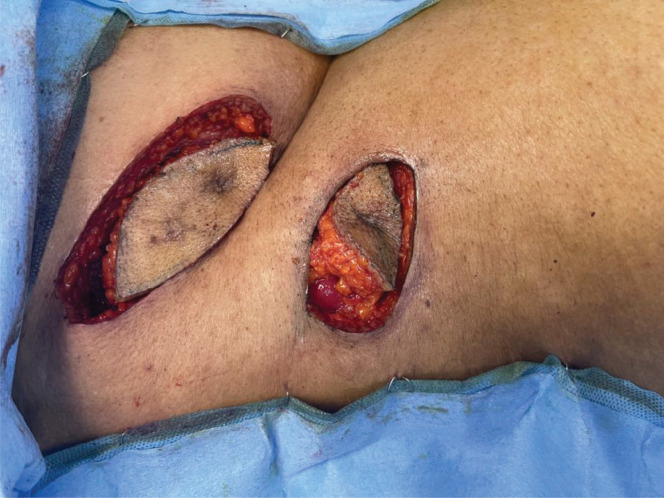
Resected skin and subcutaneous tissue around the tracts.

**Figure 7.**
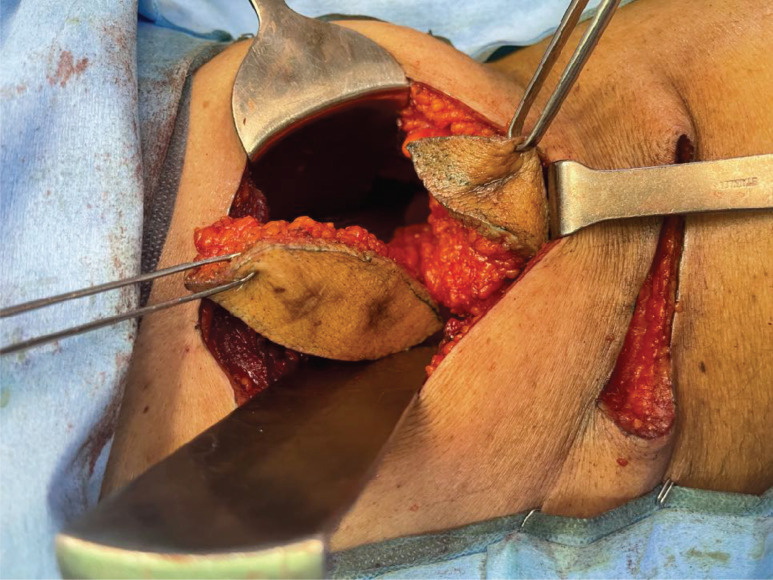
Resected tracts along with underlying abdomen muscles.

In semi prone position, the scars of PCNL were marked (Figure 5) and incised with margins. The incision was deepened to the peritoneal cavity by resecting the underlying superficial and deep abdominal muscles. Two tracts of PCNL with tumour were identified and resected (Figures 6 and 7).

#### Step 2

**Figure 8.**
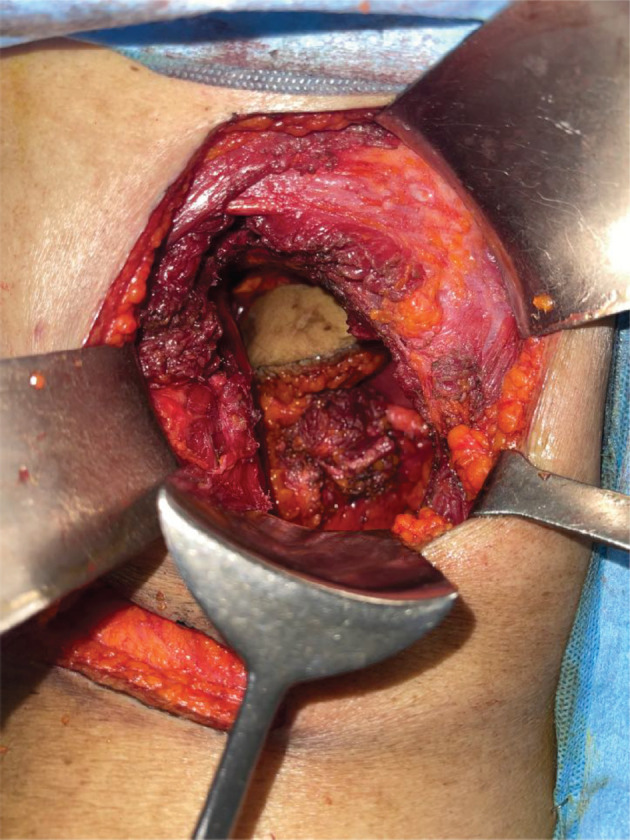
Completely resected tracts along with margins and abdomen muscles pushed into peritoneal cavity.

The specimen containing skin along with tracts of PCNL which had an oblique course were resected and pushed into the peritoneal cavity with adequate soft tissue cover all around to avoid spillage (Figure 8).

#### Step 3

**Figure 9.**
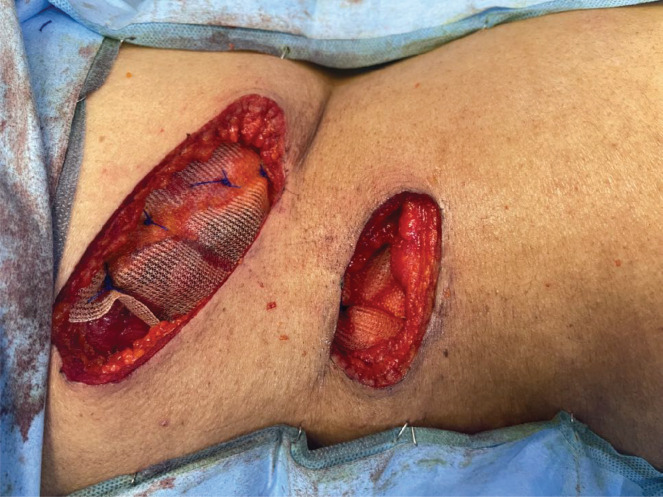
Polypropylene mesh repair for the abdomen wall defect.

**Figure 10.**
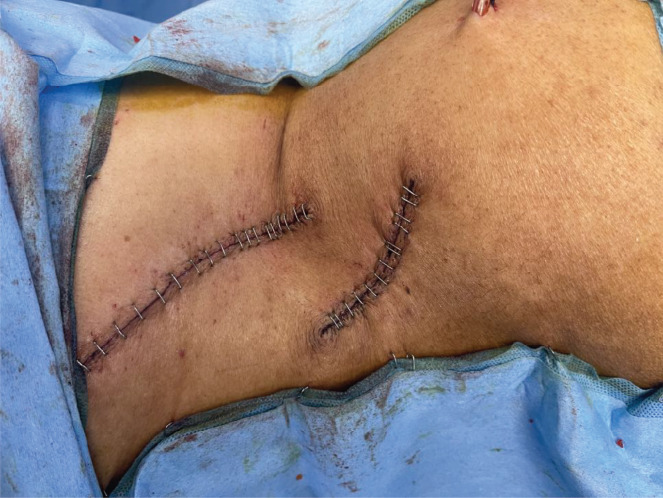
Shows resected sites after skin closure.

Mesh repair was done for both the defects and skin in order to close primarily in layers (Figures 9 and 10).

#### Step 4

**Figure 11.**
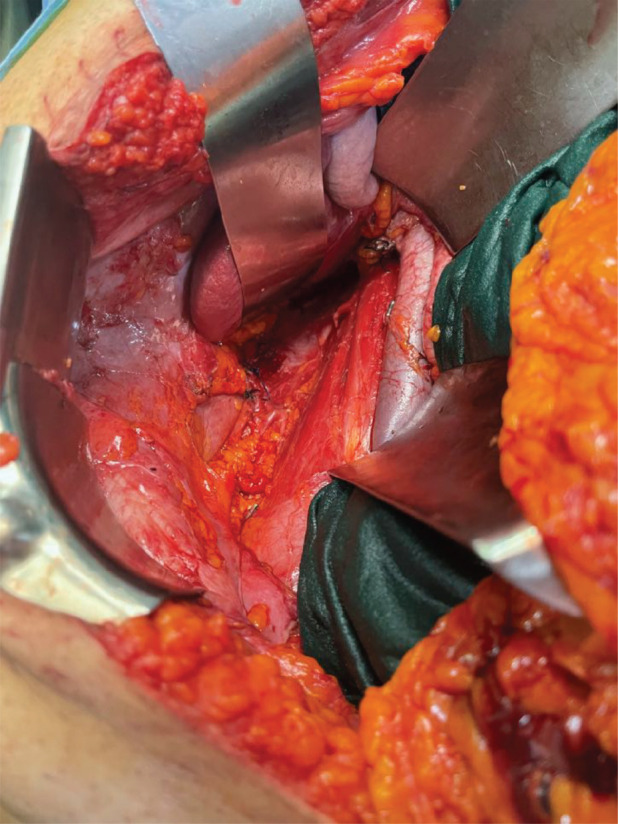
Surgical field after right radical nephroureterectomy, template-based RPLND. Resected area of tracts seen posteriorly.

The patient was turned to supine. A midline laparotomy incision was performed to access within the peritoneal cavity. Radical nephrectomy was done along with excision of tracts en masse (Figure 11).

#### Step 5

**Figure 12.**
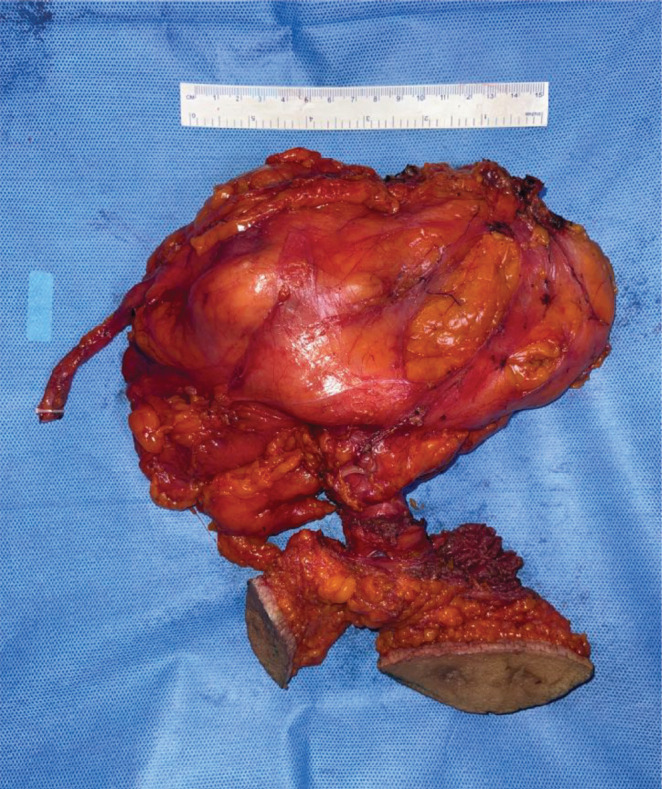
Specimen containing radical nephroureterectomy along with masse resection of PCNL tracts.

Anatomical template-based retroperitoneal lymph node dissection was done including hilar, precaval, retrocaval and inter aortocaval nodes (Figure 11). The right radical nephrectomy specimen along with resected PCNL tracts masse was represented in Figure 12.

## Discussion

SCC of the upper urinary tract is a rare entity, accounting for approximately 0.5% of renal malignant tumours and comprising 6%–15% of cancers affecting the renal pelvis and ureter [1]. Typically, it is presented between the ages of 50 to 70 years and the most common presentations include flank or abdominal pain, hematuria and abdominal mass [2]. Etiologic factors encompass nephrolithiasis, chronic inflammation, tuberculosis, azathioprine usage, chronic renal transplant rejection and prior PCNL [3]. There appears to be a temporal progression in the development of SCC from inflammation to metaplasia, dysplasia, *in situ* carcinoma and eventually carcinoma. These tumours are recognised to be aggressive with a poor prognosis, often detected at advanced stages (pT3 or greater), with less than 10% of patients surviving beyond 5 years [7, 8].

While staghorn calculus represents an established risk factor for renal pelvis SCC, the majority of cases remain undiagnosed preoperatively [4]. Conventionally, a preoperative workup for staghorn calculus involves X-ray, ultrasonography and intravenous urography, revealing findings such as filling defects, non-functional kidneys and an obstructive pattern. Suspicion for underlying SCC may arise from CT findings such as a large calculus, hydronephrosis, peri-nephric fat stranding or solid-enhancing mass lesions, although such findings on preoperative imaging are rare [9]. When a tumour is suspected, efforts to confirm the diagnosis preoperatively include urine cytology and cold cup biopsies before and during PCNL.

Radical nephrectomy is the standard of care for renal SCC, with adjuvant radiation and chemotherapy being attempted. Although PCNL is a safe procedure, the lithiasis component tends to mask the underlying SCC which disseminates after the fascial barriers in the kidney are breached. Dissemination of disease following PCNL in cases of staghorn calculus with coexisting SCC signifies a poor prognosis due to upstaging and early metastases. A surgical cure with an R0 resection offers the best outcome and extensive surgery as warranted in our case is worthwhile. Although the evidence for adjuvant therapy (radiation and chemotherapy) is weak, considering the possible tumour spread post PCNL, we offered adjuvant treatment to our patient.

To the best of our knowledge, there was only one case report of SCC of renal pelvis after PCNL presenting as integumentary neoplasm in the flank who underwent wide excision of skin mass, surrounding muscles and radical nephroureterectomy along with V-Y Gluteus Maximus myocutaneous advancement flap [3]. Our case also demonstrates a rare presentation wherein tumour was spread across the percutaneous tracts of PCNL so needed to be resected without being reported before.

## Conclusion

Long-standing staghorn calculi may serve as a harbinger for the development of SCC within the renal pelvis, however, is extremely uncommon. When tumour is suspected, CT imaging, urine cytology and intraoperative cold cup biopsy are crucial to diagnosticate. Our case further underscores the surgical challenges posed by the necessity to resect tracts along with the radical nephroureterectomy in order to offer the best possible cure rate.

## Conflicts of interest

There are no conflicts of interest.

## Funding

No funding was received for the study.

## Informed consent

Informed consent was obtained from the Patient

## Author contribution

1 - Conceived, collected data and wrote paper

1$ - Conceived, collected and analysed

2,3 - Contributed data

## Figures and Tables

**Table 1.** Reported cases of renal pelvic carcinoma after PCNL.

Reference	Age	Sex	Time between PCNL and diagnosis of SCC	Location of spread	Histology	Treatment	Followup / status
Kim *et al* [3]	54	Female	1 month	Nephrostomy of PCNL	SCC	Resection of skin mass and RN	12 months / Alive
Katz *et al* [4]	50	Male	2 weeks	Lower pole of kidney	UC	RN, Chemotherapy	19 months / Dead
Katz *et al* [4]	65	Female	Inoperable	Diaphragm	UC with sarcomatoid differentiation	None	2 months / Dead
Tsuboi *et al* [5]	66	Male	1 month	Left kidney, paraaortic node	UC with squamous differentiation	Chemotherapy	3 months / Alive
Sivaramakrishna *et al* [6]	46	Male	8 months	Kidney	SCC	RN, excision of PCNL tract, Radiation	12 months / Alive
Current report	54	Male	3 months	Lower pole of kidney and PCNL tracts	SCC	RN, Resection of PCNL tracts along with skin, RPLND, Chemotherapy, Radiation	13 months / Alive
